# In Vitro Effects of Cabazitaxel and Menadione on Cell Growth, Metabolism, and Transcriptomic Profile of Human Prostate Cancer Cell Lines

**DOI:** 10.1155/proc/4174599

**Published:** 2026-05-17

**Authors:** Ana Laura Gómez-Rosas, Mayel Chirinos, Nancy Noyola-Martínez, Mariana Segovia-Mendoza, Nayeli Torres-Ramírez, Sandra Romero-Córdoba, Lilia G. Noriega, Mitzi García-Olivares, Fernando Larrea, David Barrera

**Affiliations:** ^1^ Department of Reproductive Biology “Dr. Carlos Gual Castro”, Instituto Nacional de Ciencias Médicas y Nutrición Salvador Zubirán, Mexico City, Mexico, innsz.mx; ^2^ Department of Pharmacology, Faculty of Medicine, Universidad Nacional Autónoma de México, Mexico City, Mexico, unam.mx; ^3^ Department of Cell Biology, Faculty of Sciences, Universidad Nacional Autónoma de México, Mexico City, Mexico, unam.mx; ^4^ Department of Genomic Medicine and Environmental Toxicology, Institute of Biomedical Research, Universidad Nacional Autónoma de México, Mexico City, Mexico, unam.mx; ^5^ Department of Biochemistry, Instituto Nacional de Ciencias Médicas y Nutrición Salvador Zubirán, Mexico City, Mexico, innsz.mx; ^6^ Department of Nutritional Physiology, Instituto Nacional de Ciencias Médicas y Nutrición Salvador Zubirán, Mexico City, Mexico, innsz.mx

**Keywords:** cabazitaxel, *IL18R1*, *IL24*, menadione, microarray, prostate cancer, reactive oxygen species

## Abstract

Cabazitaxel is a second‐generation semisynthetic taxane approved for the treatment of prostate cancer. Vitamin K, an essential nutrient involved in blood coagulation and bone metabolism, has also been shown to have antineoplastic effects. However, no information is currently available regarding the combinatorial effects of cabazitaxel and menadione (VK3, a derivative of vitamin K) on prostate cancer cells. Therefore, we investigated the in vitro effects of cabazitaxel, VK3, and their combination on growth, mitochondrial bioenergetics, and glycolytic parameters using the human prostate cancer cell lines PC‐3 and DU 145. All treatments inhibited the cell growth, but the combination of cabazitaxel and VK3 produced a synergistic effect that was stronger than either compound alone, and these effects were accompanied by cell line–specific bioenergetic changes and glycolytic responses. Furthermore, high‐throughput transcriptomic profiling of PC‐3 cells revealed distinct sets of differentially expressed genes for each treatment, with the greatest effect established by the combinatorial treatment, followed by VK3, and then cabazitaxel. Gene ontology analyses showed that the combinatorial treatment was associated with biological processes such as positive regulation of reactive oxygen species metabolic process, steroid metabolic process, proteolysis, and signal transduction. Notably, the treatments altered the gene expression of several tumorigenic and immunologic mediators, including *MSXI*, *ZRSR2*, *GALNTL6*, *IL24*, and *IL18R1*, which may impact cancer cell behavior. In conclusion, these in vitro findings indicate that the combination of cabazitaxel with VK3 is more effective in inhibiting prostate cancer cell growth than either agent alone and provide exploratory mechanistic insight into their effects on cancer cell metabolism and gene expression.

## 1. Introduction

Prostate cancer (PCa) is a multifactorial etiology disease characterized by genetic and phenotypic heterogeneity [1]. Patients with PCa initially respond to androgen deprivation therapy [2, 3]. However, due to tumor heterogeneity, many patients progress to castration‐resistant prostate cancer (CRPC) that exhibits increased proliferative and invasive capabilities, leading to a more aggressive phenotype and metastasis [4]. The standard treatment for CRPC has been docetaxel; however, about 50% of patients do not respond to this therapy, and those who do eventually develop resistance [5, 6]. Cabazitaxel (CBZ) is a second‐generation semisynthetic taxane that was engineered as a dimethyloxy derivative of docetaxel. This modification enables CBZ to be effective against docetaxel‐refractory PCa, allowing it to be used as a chemotherapeutic agent for CRPC [7–10]. Generally, the taxanes impair the natural dynamics of microtubule formation, resulting in the inhibition of the mitotic cell process [8, 11]. However, similar to other antineoplastic agents, the use of high doses or prolonged treatment periods of CBZ results in undesirable side effects [6, 7]. Consequently, the development of therapeutic approaches using the combination of two or more drugs to reduce their doses is a frequent strategy for cancer treatment. In this sense, the combination of CBZ with other chemotherapeutic agents has proved an increased efficacy [11]. However, there is no information available on the effects of combinatorial treatment with CBZ and chemopreventive compounds from natural sources such as vitamins in PCa. In this context, vitamin K (VK) is a nutrient [12] with inhibitory effects on the growth of various malignancies, including PCa [13–16]. Structurally, VK has a core naphthoquinone skeleton and, depending on isoprene residues in the side chain, it is classified as phylloquinone (VK1), menaquinone (VK2), or menadione (VK3). VK1 is mainly obtained from green vegetables, VK2 is present in dairy products, meat, and eggs or produced by mammalian intestinal bacteria from VK1 [12], while VK3, considered as a synthetic form of VK, is produced in the body by metabolic conversion of VK1 [17]. Although similar at the molecular level, these agents exhibit variable bioactivity, potency, and efficacy, attributable to differences in chemical structure, enzymatic affinity, microenvironmental conditions, and cell‐specific regulation [18, 19]. Among VK congeners, VK3 exhibits the highest antineoplastic potency [20, 21]. Moreover, the combination of VK3 with other chemotherapeutic agents and vitamins, such as vitamin C or D, has shown additive and synergistic effects against various cancer cell types [20, 22–25]. VK3 has effects on cell cycle arrest, the generation of reactive oxygen species (ROS), apoptosis stimulation, and cell survival factors inhibition, among others [13, 26, 27]. However, no studies to date have examined the effects of CBZ, VK3, or their combination on cell growth, bioenergetics, metabolic parameters, or genome‐wide gene expression in PCa. The aim of the present study was to evaluate the combined effect of CBZ and VK3 on androgen‐independent PCa cell lines (PC‐3 and DU 145) growth, mitochondrial bioenergetics, and glycolytic parameters, as well as their impact on the transcriptomic profile of PC‐3 cells.

## 2. Materials and Methods

### 2.1. Reagents

RPMI 1640 medium (cat. #31800‐022), fetal bovine serum (FBS; cat. #16000044), trypsin‐EDTA (cat. #25200‐072), TRIzol reagent (cat. #15596026), penicillin–streptomycin solution (cat. #15140‐122), oligonucleotides for real‐time polymerase chain reaction (PCR), the Maxima First Strand cDNA Synthesis Kit (cat. #K1642), the Clariom D Assay (cat. #902923), and the GeneChip Hybridization Kit (cat. #900720) were obtained from Thermo Fisher Scientific (Waltham, MA, USA). TaqMan PCR Master Mix (cat. #04735536001) and TaqMan probes were purchased from Roche Applied Science and Roche Diagnostics (Basel, Switzerland). Cabazitaxel (cat. #SML2487), menadione (cat. #M5625), dimethyl sulfoxide (cat. #D2650), trichloroacetic acid (cat. #T9159), sulforhodamine B (SRB) (cat. #230162), and 2‐deoxy‐D‐glucose (cat. #D8375) were obtained from Sigma‐Aldrich (St. Louis, MO, USA). XFe96 microplates (cat. #103792‐100) and the Mitochondrial Stress Test Kit (cat. #103015‐100) were purchased from Agilent Technologies (Santa Clara, CA, USA). The Human IL‐24 DuoSet ELISA kit (cat. #DY1965) was obtained from R&D Systems (Minneapolis, MN, USA), and Hoechst 33342 (cat. #ENZ‐52401) was purchased from Enzo Life Sciences (Farmingdale, NY, USA).

### 2.2. Cell Lines and Culture Conditions

The study protocol was approved by the Human Research Ethics Committee of the Instituto Nacional de Ciencias Médicas *y* Nutrición Salvador Zubirán (No. 3520). Human PCa cell lines PC‐3 and DU 145 were used as well‐characterized models of androgen‐independent PCa. PC‐3 (ATCC, CRL‐1435TM, lot number 61777391) and DU 145 (ATCC, HTB‐81TM, lot number 61761869) were purchased from American Type Cell Culture Collection. Cell lines authenticity was checked by Short Tandem Repeat analysis, and the absence of mycoplasma was verified, as previously described [28]. Cells were seeded in RPMI‐1640 medium supplemented with 5% heat‐inactivated FBS, 100 U/mL penicillin, and 100 μg/mL streptomycin. Incubations were performed in humidified 5% CO_2_ and 95% air at 37°C.

### 2.3. Evaluation of Cell Growth

The protein‐binding dye SRB was used to assess changes in cell growth as previously described [28, 29]. Briefly, 96‐well tissue culture plates were used to seed 2,500 cells per well for incubation at 37°C for 24 h, followed by 3‐day incubations in the absence (control) or presence of CBZ (0.1, 0.25, 0.5, and 1.0 nM) or VK3 (2.5, 5, 7.5, and 10 μM). Control incubations (Ctr) were supplemented with 0.01% v/v dimethyl sulfoxide, used as vehicle for CBZ and VK3. Additionally, the combination of CBZ (0.25 nM) and VK3 (5 μM) was tested. After incubation, the cells were fixed and stained with SRB. Absorbance was measured at 510 nm for cell growth evaluation.

### 2.4. Combination Index Determination

To characterize the pharmacological effects of the treatment combinations, the inhibitory concentration required to reduce cell growth by 50% (IC50) was determined for each compound using dose–response curves using the Origin 8.0 software (OriginLab Corporation, Northampton, MA, USA). Subsequently, different combination schemes were evaluated in cell proliferation assays, and pharmacological interaction analyses were performed using the combination index (CI)–isobologram equation. CI values < 1, = 1, or > 1 indicate synergistic, additive, or antagonistic effects, respectively [30].

### 2.5. Mitochondrial Bioenergetic and Glycolytic Parameters

To evaluate the effect of treatments on the mitochondrial bioenergetic and glycolytic parameters, cells (5,000 per well) were plated in XFe96 microplates (Agilent Technologies). The next day, the cells were incubated in the absence or presence of CBZ (0.25 nM), VK3 (5 μM), or their combination for 24 h. Afterward, a mitochondrial stress test (Agilent Technologies) was performed using a Seahorse XFe96 Analyzer (Agilent Technologies) following the manufacturer’s instructions. Briefly, the cells were washed and incubated for 1 h in a CO_2_‐free incubator with XF RPMI medium pH 7.4 (Agilent Technologies) supplemented with 10 mM glucose, 1 mM pyruvate, and 2 mM glutamine. During the test, 1 μM oligomycin, 0.5 μM carbonyl cyanide‐*p*‐trifluoromethoxy phenylhydrazone (FCCP), 1 μM rotenone/antimycin A, and 50 mM 2‐deoxy‐D‐glucose were sequentially injected, and oxygen consumption rate (OCR) and extracellular acidification rate (ECAR) were measured three times before and after each injection. The resulting OCR and ECAR measurements were normalized by cell number using Hoechst nuclei dye in the Cytation1‐cell‐imaging‐multi‐mode‐reader (BioTek). Basal, ATP‐linked, and maximal respiration, proton leak, spare respiratory capacity (SRC), and nonmitochondrial respiration were calculated with the OCR values, and basal glycolysis, glycolytic capacity and reserve, and nonglycolytic acidification were calculated with ECAR values as previously described [31, 32].

### 2.6. RNA Isolation and Microarray Hybridization

Cells were seeded at a density of 150,000 per well in six‐well plates and incubated for 24 h in supplemented medium, either in the absence or in the presence of CBZ (0.25 nM), VK3 (5 μM), or their combination. Total RNA was obtained using the Trizol reagent [28, 33]. RNA recovery was estimated spectrophotometrically at 260/280 nm using a Synergy HT (Biotek).

RNA quality was checked on an Agilent BioAnalyzer 2100 (Agilent Technologies). Microarray analyses were performed at the core facility of the Instituto Nacional de Medicina Genómica (INMEGEN, Mexico). We used PC‐3 cells from three independent experiments with three technical replicates incubated in the absence (control) or presence of CBZ (0.25 nM), VK3 (5 μM), and their combination (*N* = 12). Only samples with RNA integrity number > 9.0 were further processed for RNA analysis using a Clariom D human microarray (Affymetrix, Santa Clara, USA). Hybridization, washing, and scanning procedures were performed according to the manufacturer’s guidelines.

Retrieved data were processed as previously described [28]. Briefly, the signal intensities were background corrected by RMA and normalized by the quantile algorithm with the oligo package of Bioconductor and genes were annotated with biomartR. Differential expression profiles of protein coding genes were computed with the limma package by a moderate *t*‐test and adjusted *p*‐value by the FDR method. Genes with a log fold change ± 0.6 were selected, and *p* < 0.05 were considered statistically significant.

### 2.7. Gene Ontology (GO) Analysis

GO analysis was conducted using DAVID (Database for Annotation, Visualization and Integrated Discovery; https://david.ncifcrf.gov/) with GO terms generated with the lists of differentially expressed genes (DEGs). The nomenclature of the enriched biological processes, cell components, and molecular functions included terms of the Gene Ontology Consortium. A REVIGO (reduce + visualize Gene Ontology; https://revigo.irb.hr) analysis was done to identify and avoid redundancy of the most representative GO terms, as well as for the construction of TreeMaps. Additionally, a gene set enrichment analysis (GSEA; https://www.webgestalt.org/) was performed with an FDR < 0.05 and normalized enrichment scores (NES) ≥ 2.0, followed by a leading‐edge analysis to identify common gene subsets within the selected GO terms.

### 2.8. Real‐Time PCR (qPCR)

Total RNA (2 μg) from each sample was reverse transcribed according to the manufacturer’s instructions using the Maxima First Strand cDNA Synthesis Kit. Primers and probes for qPCR amplifications were designed with the online software from Universal Probe Library Assay Design Center (Roche) (Table 1). Results were normalized against ribosomal protein L32 (*RPL32*) used as housekeeping gene. As previously described, qPCR amplifications were carried out on a LightCycler 480 II (Roche) [28, 34].

**TABLE 1:** Oligonucleotides and probes used for real‐time PCR analysis.

Gen	Left primer	Right primer	^a^Probe	Accession number
MSXI	CTT​CAC​CTG​CGT​CTC​AGT​GA	AAG​CAG​TAC​CTG​TCC​ATC​GC	78	NM_002448.3
ZRSR2	CTG​GCT​CCT​CCT​TTG​GGA​AG	GCC​TGC​TGT​AGT​AGT​CGT​CG	55	NM_005089.4
CYP1A1	TCT​TTG​GAG​CTG​GGT​TTG​AC	TGA​CCT​GCC​AAT​CAC​TGT​GT	9	AH007293.2
GALNTL6	CCA​GCG​ACC​CTT​TTG​AGT​CT	GCC​ACC​CAA​TTC​CCA​AAA​CC	19	NM_001034845.3
IL24	TGC​TTC​TCT​GGA​GCC​AGG​TA	AAC​AAC​CCC​CTT​CAC​TTG​GC	81	NM_006850.3
IL18R1	AGT​TCC​GGT​TCT​TCT​TGG​ACC	AAG​CAG​AGC​AGT​TGA​GCC​TT	86	NM_001371419.1
RPL32	GAA​GTT​CCT​GGT​CCA​CAA​CG	GAG​CGA​TCT​CGG​CAC​AGT​A	17	NM_000994.3

^a^From the universal probe library (Roche).

### 2.9. Detection

The PC‐3 and DU 145 cells were seeded into six‐well plates at a density of 150,000 cells per well. After 24 h, the medium was replaced with fresh medium supplemented with CBZ (0.25 nM), VK3 (5 μM), or their combination. The supernatants were collected and the IL‐24 concentration was analyzed with the Human IL24 DuoSet ELISA kit, following the manufacturer’s guidelines. The optical density of each well was determined using a microplate reader (Multiskan MS photometer type 352, Labsystems). The IL‐24 concentrations were calculated with a standard curve constructed using recombinant IL‐24. The detection range of the assay was 62.5–4,000 pg/mL.

### 2.10. Statistical Analysis

The results are expressed as the mean ± S.D. Statistical differences were determined by one‐way ANOVA, followed by appropriate post hoc tests (Holm–Sidak method for pairwise comparisons), using a specialized software package (SigmaPlot 11.0, Jandel Scientific). Differences were considered statistically significant at *p* < 0.05.

## 3. Results

### 3.1. Effects of VK3, CBZ, and Their Combination on Cell Growth of Cell Lines

The in vitro evaluation of the PC‐3 and DU 145 cell lines’ growth was analyzed in the presence of different concentrations of CBZ and VK3. As shown in Figures 1(a), 1(b), both compounds inhibited cell proliferation in a dose‐dependent manner. The IC50 values for CBZ in the PC‐3 and DU 145 cell lines were 0.27 and 0.24 nM, respectively, whereas for VK3, they were 5.22 and 5.69 μM, respectively. These concentrations were close to the lowest concentrations at which significant differences were observed. Thus, we used 0.25 nM for CBZ and 5 μM for VK3 for further experiments. Remarkably, the combination of CBZ + VK3 resulted in higher inhibition of growth in comparison to what was observed with the individual treatments (*p* < 0.05) (Figure 1(c)).

FIGURE 1Effect of CBZ and VK3 on cell growth of prostate cancer cell lines. PC‐3 (black bars) and DU 145 (white bars) were incubated for 3 days with different concentrations of (a) CBZ and (b) VK3. (c) Effect of CBZ [0.25 nM], VK3 [5 μM], and their combination. *N* > 7 independent experiments performed in sextuplicate. Data were normalized to controls without treatments, set at 100% for each experiment. ^∗^ *p* < 0.05 vs control (Ctr); ^∗∗^ *p* < 0.05 vs each compound alone.

**Figure:**
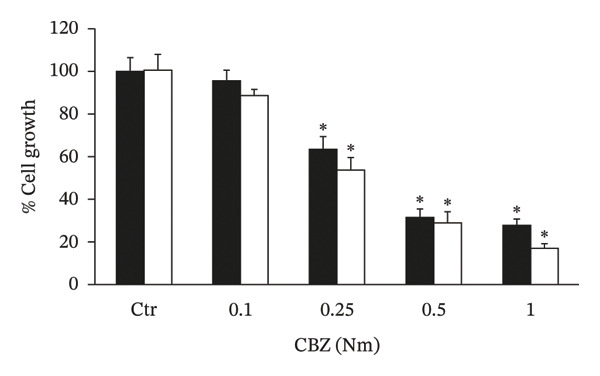
(a)

**Figure:**
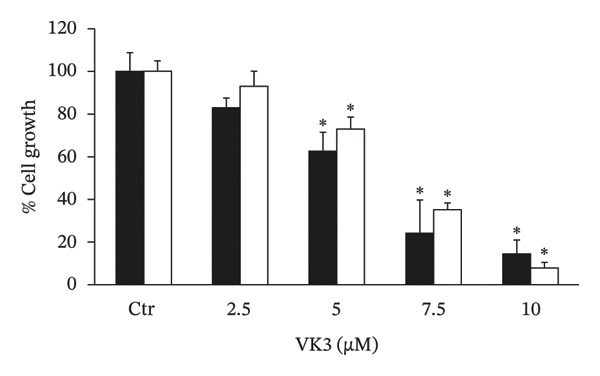
(b)

**Figure:**
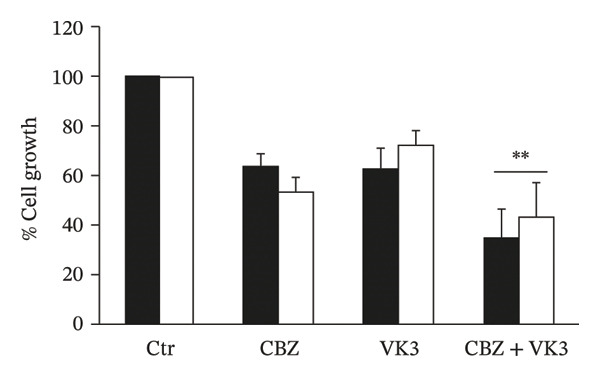
(c)

### 3.2. Combination Index Determination

To discern the pharmacological interaction of CBZ with VK3 in both cell lines, we used the combination index equation. For PC‐3 and DU 145 cells, the co‐treatment schemes (IC50/IC50) showed combination indexes of 0.66 and 0.83, respectively, indicating a synergic interaction.

### 3.3. Mitochondrial Bioenergetic and Glycolytic Parameters

To determine the effect of CBZ, VK3, and their combination on the mitochondrial bioenergetics and glycolytic parameters, we evaluated mitochondrial respiration, measured by OCR, and glycolytic activity, measured by ECAR (Figures 2 and 3). Both parameters were altered in PC‐3 and DU 145 cells under the different treatments, reflecting impaired mitochondrial activity and glycolytic adaptability.

FIGURE 2Oxygen consumption rate (OCR) in PC‐3 (a) and DU 145 (b). Mitochondrial bioenergetic parameters in PC‐3 and DU 145 in the absence or presence of CBZ [0.25 nM], VK3 [5 μM], or their combination at 24 h. Left panels: Representative time course of OCR before and after the injection of oligomycin (O), carbonyl cyanide‐*p*‐trifluoromethoxy phenylhydrazone (F), rotenone/antimycin A (R/A), and 2‐deoxy‐D‐glucose (2DG). Right panels: Calculated parameters of mitochondrial bioenergetics. Data are expressed as the mean ± SEM. The figure is representative of three biological replicates, each performed with six technical replicates per treatment, and analyzed using a *t*‐test. ^∗^ *p* < 0.05 vs Ctr.

**Figure:**
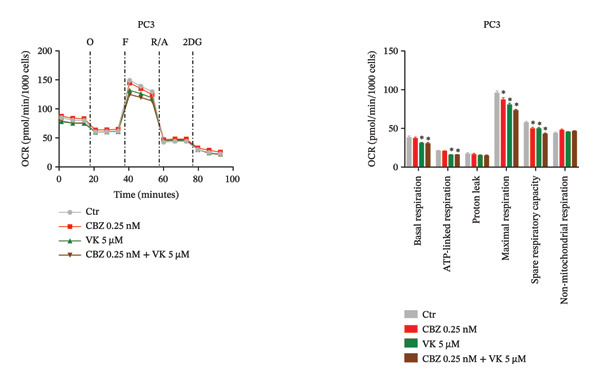
(a)

**Figure:**
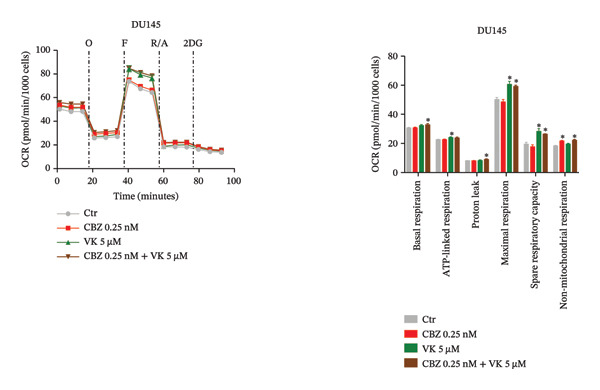
(b)

FIGURE 3Extracellular acidification rate (ECAR) in both cancer lines, PC‐3 (a) and DU 145 (b). Glycolytic parameters in PC‐3 and DU 145 (a and b), with Ctr (untreated cell) and treatment at 24 h. Left panels: Representative time course of ECAR before and after the injection of oligomycin (O), carbonyl cyanide‐*p*‐trifluoromethoxy phenylhydrazone (F), rotenone/antimycin A (R/A), and 2‐deoxy‐D‐glucose (2DG). Right panels: calculated glycolytic parameters. Data are expressed as the mean ± SEM. The figure is representative of three biological replicates, each performed with six technical replicates per treatment, and analyzed using a *t*‐test. ^∗^ *p* < 0.05 vs Ctr.

**Figure:**
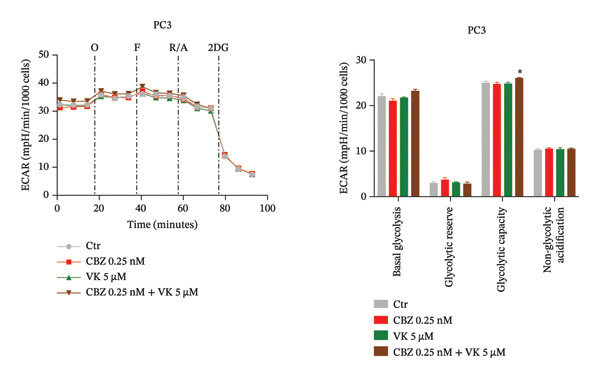
(a)

**Figure:**
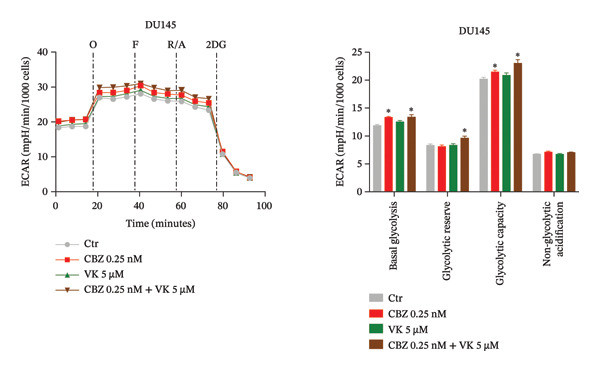
(b)

Notably, in PC‐3 cells, VK and the combinatorial treatment reduced basal respiration and ATP‐linked respiration, while CBZ, VK, and their combination reduced both the maximal respiration and SRC, indicating a reduced ability of these cells to cope with metabolic stress (Figure 2(a)). In contrast, DU 145 cells showed increased maximal respiration and SRC in response to VK3 and combinatorial treatment as well as elevated basal respiration and proton leak specifically with the combination (Figure 2(b)). Nonmitochondrial respiration showed only minor variations in both cell lines, reaching statistical significance exclusively in DU 145 cells treated with CBZ or the combination (Figures 2(a), 2(b)).

Regarding the glycolytic function, the effects on ECAR were cell line–dependent (Figure 3). Interestingly, the combinatorial treatment showed an increased glycolytic capacity in both cell lines, while significant changes in the basal glycolysis and the glycolytic reserve were observed only in DU 145 cells. In addition, CBZ increased basal glycolysis and glycolytic capacity in DU 145 cells (Figures 3(a), 3(b)). Nonglycolytic acidification remained largely unchanged in both cell lines.

### 3.4. Transcriptomic Changes Induced by Treatments on PC‐3 Cell Line

To get a deeper overview of the molecular mechanisms involved in the cell growth inhibitory effects of CBZ and VK3, alone or in combination, we investigated their effects on gene expression of PC‐3 cells. A microarray analysis revealed different sets of DEGs (logFC: ± 0.6, *p* < 0.05) induced by the treatments. Overall, the larger list of DEGs was identified with the combinatory treatment (CBZ + VK3, *N* = 132), followed by VK3 (*N* = 112) and CBZ (*N* = 66) (Figure 4(a). Opposed to VK3 effect, transcriptional effects of CBZ and the combinatorial treatment mostly resulted in the down‐modulation of DEGs (Figure 4(a)). A comparative analysis of the transcriptomic profiles revealed that most DEGs were exclusive to each treatment, and although few genes overlapped between the different conditions, none overlapped simultaneously for the three conditions (Figure 4(b)). Specifically, we only found 11 overlapped upregulated DEGs between VK3 and CBZ + VK3 (Figure 4(c)), while for downregulated DEGs, there were 5 common DEGs between CBZ and VK3, 11 between CBZ and CBZ + VK3, and 11 between VK3 and CBZ + VK3 (Figure 4(d), Supporting Table S1).

FIGURE 4Overview of DEGs modulated by CBZ, VK3, and their combination. (a) Bar plot showing the total number of upregulated and downregulated genes induced by the treatments vs control (untreated). Venn diagrams showing the overlapping of (b) total, (c) upregulated, and (d) downregulated DEGs across treatments.

**Figure:**
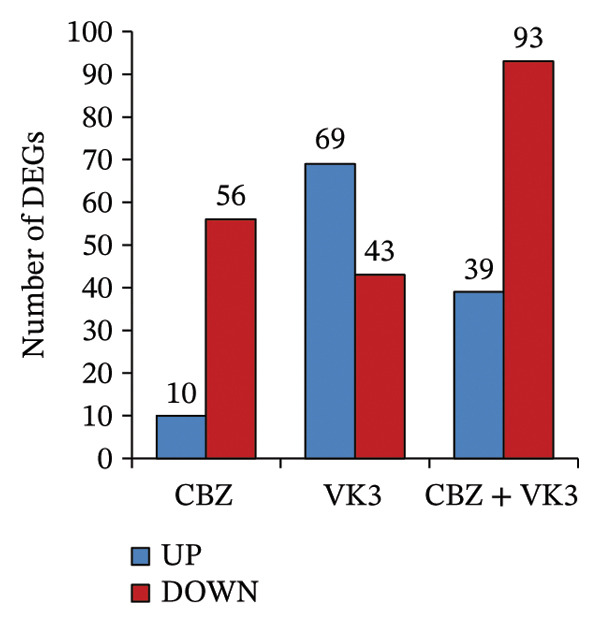
(a)

**Figure:**
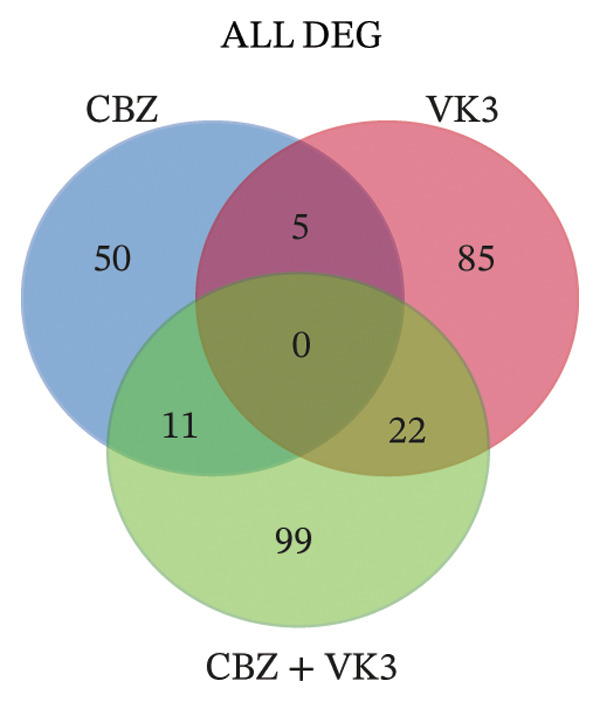
(b)

**Figure:**
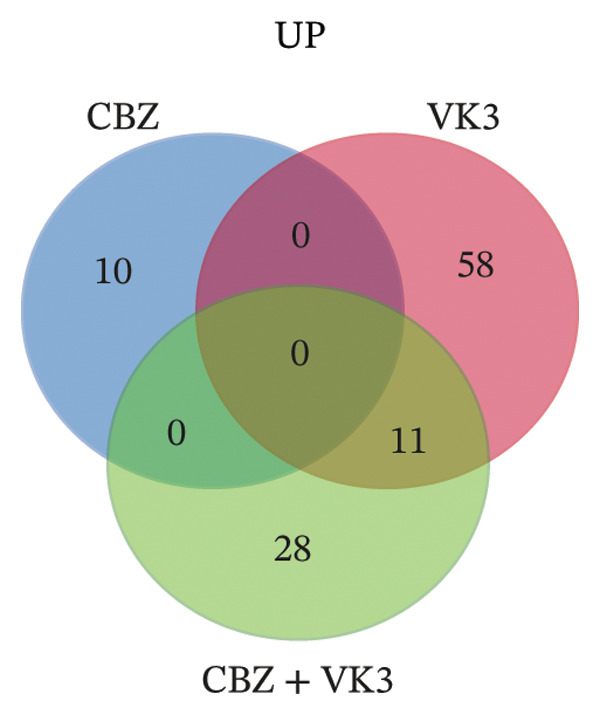
(c)

**Figure:**
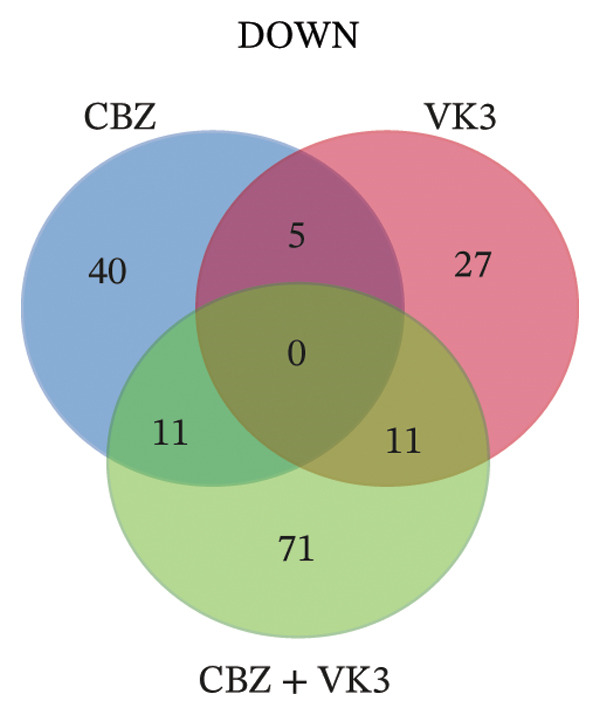
(d)

### 3.5. GO Analysis of the DEGs

We used different approaches to ascertain the most relevant biological processes related with the expression profile changes established by each treatment. GO analysis followed by REVIGO highlighted key biological processes altered by each treatment (Table 2). CBZ effects are associated with neutrophil chemotaxis and the regulation of the ERK1 and ERK2 cascades. In relation to VK3, we found that the major transcriptomic changes were associated with steroid metabolic processes, positive regulation of interleukin‐1 beta production, and positive regulation of ROS metabolic processes, as well as negative regulation of cell population proliferation. Moreover, the combinatorial treatment promoted expression changes in genes involved in the positive regulation of ROS metabolic processes, cellular response to organic cyclic compound, signal transduction, and proteolysis, among others. REVIGO TreeMaps were further constructed to summarize the findings (Figure 5).

**TABLE 2:** Top biological processes identified by REVIGO in CBZ, VK3, and CBZ + VK3 *vs* control.

Contrast	GO ID	Description	Value	LogSize
CBZ	0043584	Nose development	−1.706	2.79
	0030593	Neutrophil chemotaxis	−1.634	3.43
	0070374	Positive regulation of ERK1 and ERK2 cascade	−1.570	4.15

VK3	0042448	Progesterone metabolic process	−4.155	3.45
	0097267	Omega‐hydroxylase P450 pathway	−2.824	1.94
	0021702	Cerebellar Purkinje cell differentiation	−2.754	2.81
	0008202	Steroid metabolic process	−2.733	5.13
	0008285	Negative regulation of cell population proliferation	−2.278	4.59
	1904681	Response to 3‐methylcholanthrene	−2.013	1.28
	0030198	Extracellular matrix organization	−1.994	4.82
	2000379	Positive regulation of reactive oxygen species metabolic process	−1.926	3.30
	0071407	Cellular response to organic cyclic compound	−1.878	4.68
	0010041	Response to iron(III) ion	−1.838	3.55

CBZ + VK3	0042448	Progesterone metabolic process	−3.876	3.45
	2000379	Positive regulation of reactive oxygen species metabolic process	−2.963	3.30
	0097267	Omega‐hydroxylase P450 pathway	−2.648	1.94
	0006508	Proteolysis	−2.217	6.30
	0050868	Negative regulation of T cell activation	−2.160	3.99
	0034276	Kynurenic acid biosynthetic process	−1.924	1.85
	1904681	Response to 3‐methylcholanthrene	−1.924	1.28
	0008210	Estrogen metabolic process	−1.806	3.49
	0071407	Cellular response to organic cyclic compound	−1.709	4.68
	0071395	Cellular response to jasmonic acid stimulus	−1.625	3.73

FIGURE 5TreeMaps of the biological processes constructed by REVIGO summarizing the over‐represented biological processes. (a) VK3 and (b) combinatorial treatment (CBZ + VK3) vs control contrasts. Categories with *p* < 0.05 were used to generate the TreeMaps, colored by functional category. The size of the boxes is proportional to the *p*‐values of each category.

**Figure:**
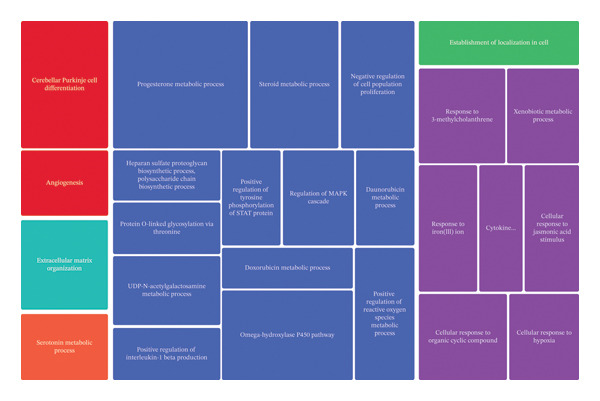
(a)

**Figure:**
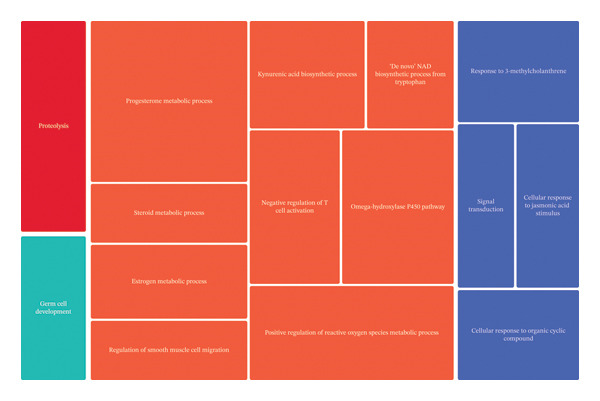
(b)

Furthermore, the enrichment pathway analysis by GSEA revealed that CBZ effects on PC‐3 cell line are associated with biological processes such as the regulation of the MAPK cascade (Figure 6(a)). For VK3 and combinatorial treatments, GSEA analysis showed similar results, highlighting biological processes linked to icosanoid, fatty acid, and alcohol metabolic processes as well as secondary and cellular ketone metabolic processes, among others (Figures 6(b), 6(c)). Moreover, the combinatorial treatment showed a higher enrichment score for positive regulation of ROS metabolism than VK3 alone. Additionally, we used the Enrichr tool to construct bar plots and clustergrams of over‐represented biological processes and pathways (KEGG) (Supporting Figure S1). Furthermore, the biological processes, cellular components, and molecular functions retrieved for each treatment are summarized in Supporting Figure S2.

FIGURE 6Gene set enrichment by GSEA. Bar plots of the biological processes enriched in (a) CBZ, (b) VK3, and (c) CBZ + VK3. *p* < 0.05.

**Figure:**
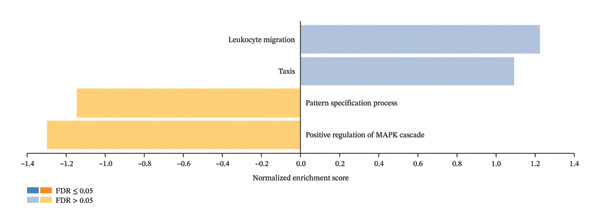
(a)

**Figure:**
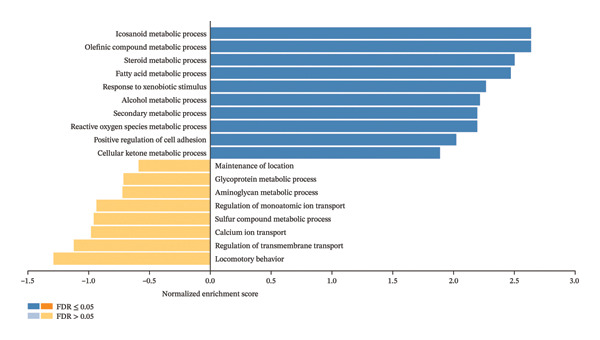
(b)

**Figure:**
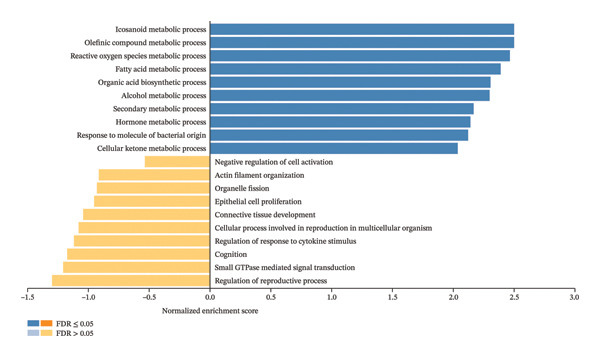
(c)

### 3.6. Validation of DEGs

To validate the DEGs identified in the microarray analysis, we selected several genes from each contrast among those with the highest fold changes that has previously been reported to be deregulated in various human malignant neoplasms. For CBZ contrast validation, qPCR quantification of *MSX1* (msh homeobox 1) and *ZRSR2* (zinc finger CCCH‐type, RNA binding motif and serine/arginine rich 2) showed that these genes were up‐ and downregulated, respectively (Figure 7(a)). Likewise, for VK3, we assessed the differential expression of *CYP1A1* (Cytochrome P450 Family 1 Subfamily A Member 1) and *GALNTL6* (Polypeptide N‐Acetylgalactosaminyltransferase Like 6) that were up‐ and downregulated, respectively (Figure 7(b)). For the combinatorial treatment, we corroborated the upregulation of *IL24* (interleukin 24) and *IL18R1* (interleukin 18 receptor 1) (Figure 7(c)). In all cases, we observed significant differential expression of these genes in PC‐3, consistent with the directionality indicated by the microarray analysis (Supporting Table S2). Interestingly, the DU 145 cell line also exhibited significant differential expression with same directionality for most of the assessed genes, although no significant differences were detected for *CYP1A1* and *IL18R1*.

FIGURE 7Independent validation of selected DEGs from the microarray analysis. qPCR was used to evaluate the differential expression of up‐ and downregulated genes in cells treated with (a) CBZ [0.25 nM], (b) VK3 [5 μM], and (c) the combination CBZ + VK3. Black bars: PC‐3, white bars: DU 145. Each bar represents the mean ± S.D. of three independent measurements. qPCR data were normalized against RPL32 gene expression. Values for the control were set to 1. ^∗^ *p* < 0.05 vs control (Ctr).

**Figure:**
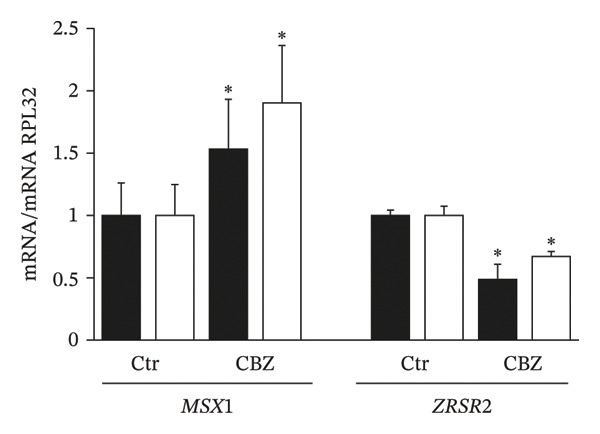
(a)

**Figure:**
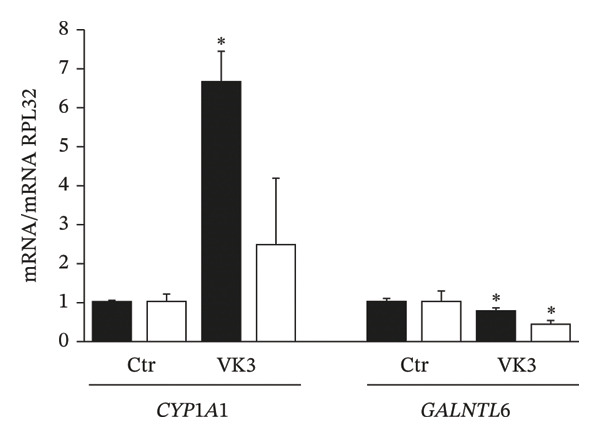
(b)

**Figure:**
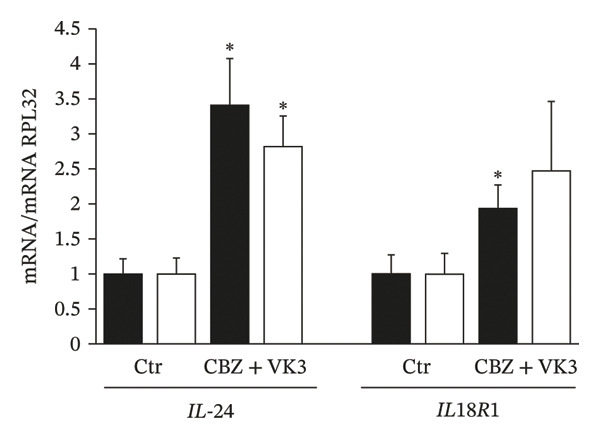
(c)

### 3.7. Secretion of IL‐24 Protein

**FIGURE 8:**
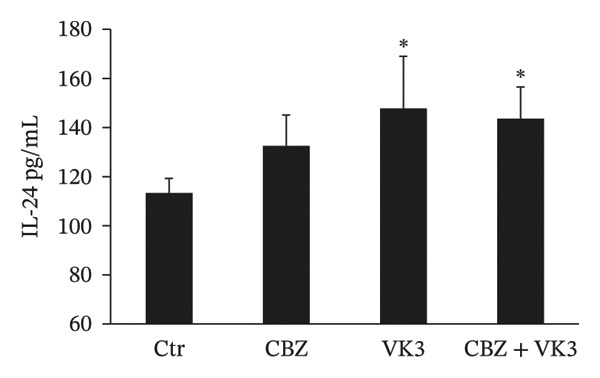
IL‐24 secretion modulated by CBZ, VK3, and their combination in PC‐3 cell line. IL‐24 concentration was quantified in cell culture media after treatments in the absence or presence of CBZ [0.25 nM], VK3 [5 μM], or their combination. Each bar represents the mean ± S.D. of three independent measurements. Results are expressed as pg/mL. ^∗^ *p* < 0.05 vs control (Ctr).

To further assess the biological significance of the transcriptional changes observed, *IL24* gene expression at the protein level was validated by ELISA. The results showed that IL‐24 secretion was significantly stimulated by VK3 and the combinatorial treatment in PC‐3 cells (Figure 8). However, for DU 145 cells, VK3 and the combinatorial treatment exhibited an upward trend, but changes were not statistically significant (data not shown).

## 4. Discussion

In standard cancer therapy, most cancer cells develop chemotherapeutic resistance and/or the patients suffer undesirable adverse side effects. These disadvantages have fueled efforts to develop novel anticancer strategies to reduce toxicity to normal cells by using the combination of two or more treatments [6, 35]. Accordingly, the combination of antineoplastic agents with chemopreventive compounds derived from natural sources has been explored, as it may potentially delay or reduce cancer progression while minimizing side effects. In this regard, several studies have shown that vitamins can sensitize diverse cancer cell lines by mechanisms that include cell cycle arrest, apoptotic events, cell survival factors inhibition, induction of tumor suppressor genes, and generation of ROS, among others [23, 27].

In the present study, we observed that CBZ and VK3 inhibited cell growth in PC‐3 and DU 145 cell lines, in agreement with their well‐documented antiproliferative effects in various cancer cell lines [9, 11, 14, 15, 36]. Notably, we demonstrated for the first time that the combination of CBZ with VK3 produces a synergistic interaction to inhibit cell growth, exhibiting greater suppression of proliferation than either agent alone and highlighting its potential as a therapeutic strategy with clinical relevance.

Cancer cells frequently remodel their mitochondrial bioenergetics and glycolytic programs to sustain growth, survival, and metastasis [37]. Our data suggest that the antiproliferative effects of CBZ, VK3, and especially their combination arise in part from disrupting these metabolic programs, potentially through mitochondria‐specific redox cycling that exploits intrinsic mitochondrial vulnerabilities. Unexpectedly, PC‐3 and DU 145 exhibited dissimilar responses, indicating that the effects of the treatments are cell line–dependent. In PC‐3 cells, the pronounced reduction in basal and maximal respiration, accompanied by a decrease in SRC, suggests a profound impairment of mitochondrial function and a loss of metabolic flexibility—conditions that may render these cells more vulnerable to stress‐induced cell death. Conversely, DU 145 cells exhibited the opposite trend, showing increased maximal respiration and SRC, indicative of compensatory mechanisms aimed at maintaining energy homeostasis or the biosynthesis of nucleotides. Assessment of glycolytic activity via ECAR revealed additional cell‐specific patterns. The combinatorial treatment increased glycolytic capacity in both PC‐3 and DU 145 cells, suggesting a compensatory reliance on glycolysis under mitochondrial inhibition. In DU 145 cells, basal glycolysis and glycolytic reserve were also significantly elevated, and CBZ alone further enhanced glycolytic activity, underscoring a higher metabolic flexibility in these cells. Nonglycolytic acidification remained largely unaffected, confirming the specificity of these effects. Collectively, these findings highlight that PC‐3 cells are particularly vulnerable to mitochondrial perturbation induced by VK3 and combination therapy, whereas DU 145 cells exhibit adaptive metabolic rewiring, engaging both mitochondrial and glycolytic pathways to maintain energetic homeostasis. This divergent response reflects the metabolic heterogeneity of PCa cells [1, 38]. A notable difference between these cell lines is their p53 status: PC‐3 cells lack functional p53 due to a deletion in the *TP53* gene, whereas DU 145 cells harbor two missense mutations in this gene [39]. Beyond its canonical cellular functions, p53 also plays a pivotal role in the regulation of cellular metabolism. Loss of p53 promotes metabolic reprogramming, shifting cells from an oxidative phosphorylation (OXPHOS)‐dependent phenotype toward a glycolysis‐driven metabolic state. In PCa, previous studies have shown that amino acid restriction—particularly glutamine deprivation—enhances glucose consumption in DU 145 cells through a p53‐dependent mechanism [1, 40]. The complementary p53 status makes the two models particularly valuable for investigating PCa progression and therapy response, thereby enhancing the translational relevance of these findings to CRPC.

To further investigate the molecular basis underlying PCa cell growth inhibition, we performed a transcriptomic profiling and GO analysis induced by each treatment on PC‐3 cells, a more aggressive PCa cell line compared to DU 145. GO analysis indicated that VK3 and the combinatorial treatment affected ROS metabolic processes, consistent with previous reports demonstrating that both VK and CBZ can promote ROS production [14, 20, 41–43]. Oxidative stress plays a critical role in various physiological and pathological processes, including cancer, and evidence suggests that cancer cells are more vulnerable than normal cells to ROS‐induced damage by exogenous agents, which can ultimately lead to cell death [24, 44]. In line with this, our microarray analysis revealed upregulation of ROS‐producing genes such as *CYP1A1*, *CYP1B1*, and *IL24* [45–47], along with downregulation of the protective gene *HSPB8* [48], supporting a transcriptional program that favors cell death.

Overall, our results indicate that disruption of the ROS balance contributes to the suppressive effects of VK3, CBZ, and their combination on PCa cell growth. However, it is unlikely to be the only mechanism involved, as previous studies have shown that both agents also exert antitumor activity through additional pathways [9, 23, 44]. Accordingly, we identified and validated several genes previously implicated in cell growth inhibition, acting as growth suppressors, negative regulators of the cell cycle, or by inducing mechanisms that halt tumor cell proliferation, whose dysregulation has been associated with various malignancies. In this context, we demonstrated for the first time that CBZ modulated two mediators of tumorigenesis, *MSX1* and *ZRSR2*, which were up‐ and downregulated, respectively. Interestingly, the overexpression of *MSX1* has been reported to inhibit proliferation and migration in cervical cancer cells [49]. Furthermore, ectopic *MSX1* expression significantly suppressed the clonogenicity, proliferation, migration, and invasion in breast tumor cells by inducing both cell cycle arrest and apoptosis [50]. In contrast, He et al. reported that *ZRSR2* is significantly upregulated in CRPC tumors. Moreover, knockdown of *ZRSR2* reduced PCa cell proliferation and delayed cell cycle progression, correlating elevated *ZRSR2* expression with poor clinical outcomes [51]. Additionally, *ZRSR2* knockdown in HeLa cells decreased cell viability, and its downregulation caused splicing defects, impaired in vitro clonogenic ability, and suppressed tumor formation in mice [52]. Further research is needed to determine whether *MSX1* and *ZRSR2* play similar roles in PCa.

Regarding VK3, we validated *CYP1A1* and *GALNTL6* as responsive genes. *CYP1A1* encodes a cytochrome P450 enzyme involved in the metabolism of fatty acids, steroid hormones, and vitamins. Interestingly, *CYP1A1* is upregulated in PCa cell lines and tissues, and its knockdown inhibits cell growth in a time‐dependent manner [53]. However, in vivo investigations have shown that certain natural dietary chemopreventive compounds activate CYP1A1 [54]. Therefore, further investigations are required to clarify the role of *CYP1A1* in cancer regulation.

On the other hand, *GALNTL6* was downregulated in the presence of VK3. In this sense, the upregulation of acetylgalactosyltransferase genes and/or their malfunction have been associated with tumor progression [55]. *GALNTL6*, which mediates O‐linked glycosylation of proteins via threonine residues, is amplified in papillary thyroid carcinomas, and its overexpression correlates with poor prognosis and reduced survival in cervical and thyroid cancers [56–58]. Therefore, the observed downregulation of *GALNTL6* may contribute to the inhibition of cell proliferation.

Lastly, the combination of CBZ with VK3 induced the expression of *IL18R1* and *IL24*. *IL18R1* encodes for a cytokine receptor that specifically binds to IL18/IL37, thereby exerting biological effects. Interestingly, *IL18R1* overexpression inhibits the growth and migration of cancer cells in lung squamous cell carcinoma [59] and has been proposed as a potential immunological marker in triple‐negative breast cancer [60, 61]. In relation to IL‐24, initially known as the melanoma differentiation–associated gene‐7 (MDA‐7) protein, it is considered a tumor suppressor cytokine involved in the inhibition of invasion and metastasis in a wide variety of solid tumors [62–64]. IL‐24 levels decline during cancer progression, and it is very low in metastatic stages. Moreover, MDA‐7/IL‐24 has demonstrated potent anticancer activity in preclinical studies and phase I clinical trials in patients with advanced cancers [65]. Functionally, it has been reported that IL‐24 increases the ROS in cancer cells, and this increase may generate oxidative stress ultimately leading to cell death [31, 46]. Further investigation is warranted to fully elucidate IL‐24’s contribution to the response of cancer cells treated with CBZ and VK3.

While these transcriptomic changes provide mechanistic insight, a limitation of this study is that transcriptomic profiling was performed only in PC‐3 cells. When selected genes were validated in PC‐3 and DU 145 models, some showed comparable expression changes, whereas others exhibited only nonsignificant trends in DU 145 cells, likely reflecting intrinsic cellular heterogeneity. Given the differences between these cell lines, the effects of CBZ and VK3 on transcriptomic changes of DU 145 should be further investigated.

Collectively, the findings suggest that CBZ and VK3 inhibit PCa cell growth by impairing mitochondrial resilience, promoting oxidative stress, and modulating tumor‐related genes. The divergent metabolic responses in each cell line highlight the importance of tumor diversity in shaping treatment outcomes and underscore the potential of exploiting metabolic vulnerabilities to enhance therapeutic efficacy.

## 5. Conclusion

The present study demonstrates that CBZ combined with VK3 produces synergistic antiproliferative effects in PCa cells. This activity is associated with impairing mitochondrial resilience, increased oxidative stress, upregulation of tumor‐suppressive mediators (*MSX1, IL24*), and downregulation of oncogenic factors (*ZRSR2, GALNTL6*). These changes reveal redox‐driven and metabolic vulnerabilities that compromise cancer‐cell survival. Cell line–specific differences in mitochondrial bioenergetic responses highlight the metabolic heterogeneity of PCa and suggest that tumors with limited metabolic flexibility may be particularly susceptible to CBZ + VK3.

Future investigations should confirm these findings in vivo, define the mechanistic contribution of ROS‐related and metabolic pathways, and identify biomarkers predictive of therapeutic response. Such studies may support the integration of VK as an adjuvant to CBZ, potentially enabling dose reduction and enhancing efficacy in the treatment of PCa.

## Funding

This work was supported partially by the Annual Acquisition Program of the Department of Reproductive Biology at the INCMNSZ (BRE‐3520 to David Barrera) and PAPIIT‐DGAPA‐UNAM (grant number IA204723).

## Disclosure

This article is part of the doctoral thesis of Ana Laura Gómez‐Rosas, who is a Ph.D. student from Programa de Doctorado en Ciencias Biológicas, Universidad Nacional Autónoma de México (UNAM), supported with fellowship number 762461 from Secretaría de Ciencia, Humanidades, Tecnología e Innovación (SECIHTI, Mexico). In addition, Nancy Noyola‐Martínez received a postdoctoral fellowship from the Patronage of the National Institute of Medical Sciences and Nutrition Salvador Zubirán and Fundación para la Salud y la Educación Salvador Zubirán, A.C. (FunSaEd), México.

## Conflicts of Interest

The authors declare no conflicts of interest.

## Supporting Information

Additional supporting information can be found online in the Supporting Information section.

## Supporting information


**Supporting Information 1** Supporting Figure S1. Functional analysis of DEG. Bar plots and clustergrams of over‐represented BPs (left, GO Biological Processes) and pathways (right, KEGG), retrieved by Enrichr; A) VK3 and (B) CBZ + VK3.

**Supporting Information 2** Supporting Figure S2. GO terms for Biological Processes. Cellular components and molecular functions are represented by red, blue, and green bars, respectively. A) CBZ, B) VK3, and C) CBZ + VK3. The height of the bar represents the number of IDs in the user list and also in the category.

**Supporting Information 3** Overlapped DEGs in the different contrasts.

**Supporting Information 4** Supporting Table S2. Selected DEGs from microarray analysis of CBZ, VK3, and CBZ + VK3 versus CTR. DEG: differentially expressed genes; FC: fold change.

**Supporting Information 5** List of DEGs from microarray analysis.

## Data Availability

Raw data from microarray analysis were generated in a core facility, but all processed data are available from the authors.
