# Supplementary material for: In Vitro Effects of Cabazitaxel and Menadione on Cell Growth, Metabolism, and Transcriptomic Profile of Human Prostate Cancer Cell Lines
Source: Prostate Cancer. 2026 May 17;2026:4174599. doi: 10.1155/proc/4174599 (PMC13181216; doi:10.1155/proc/4174599)
Supplement: Supplementary file 1 — Supporting Information 1 Supporting Figure S1. Functional analysis of DEG. Bar plots and clustergrams of over‐represented BPs (left, GO Biological Processes) and pathways (right, KEGG), retrieved by Enrichr; A) VK3 and (B) CBZ + VK3. [file PROC-2026-4174599-s001.pdf]

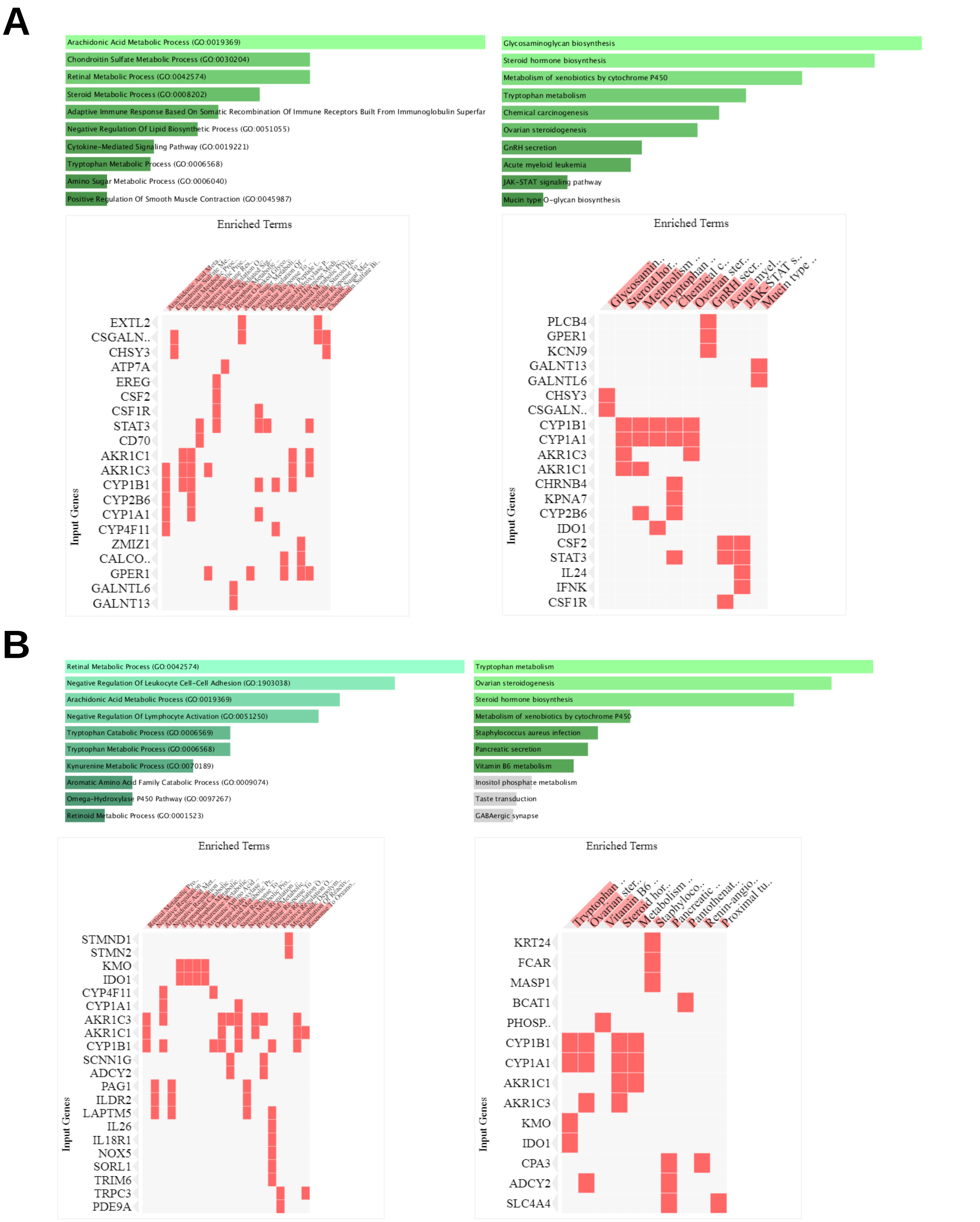

S1 Fig. Functional analysis of DEG. *Bar plots and clustergrams of overrepresented BPs (left, GO Biological Processes) and pathways (right, KEGG), retrieved by Enrichr; A) VK3 and (B) CBZ+VK3.*
