# Supplementary material for: In Vitro Effects of Cabazitaxel and Menadione on Cell Growth, Metabolism, and Transcriptomic Profile of Human Prostate Cancer Cell Lines
Source: Prostate Cancer. 2026 May 17;2026:4174599. doi: 10.1155/proc/4174599 (PMC13181216; doi:10.1155/proc/4174599)
Supplement: Supplementary file 2 — Supporting Information 2 Supporting Figure S2. GO terms for Biological Processes. Cellular components and molecular functions are represented by red, blue, and green bars, respectively. A) CBZ, B) VK3, and C) CBZ + VK3. The height of the bar represents the number of IDs in the user list and also in the category. [file PROC-2026-4174599-s002.pdf]

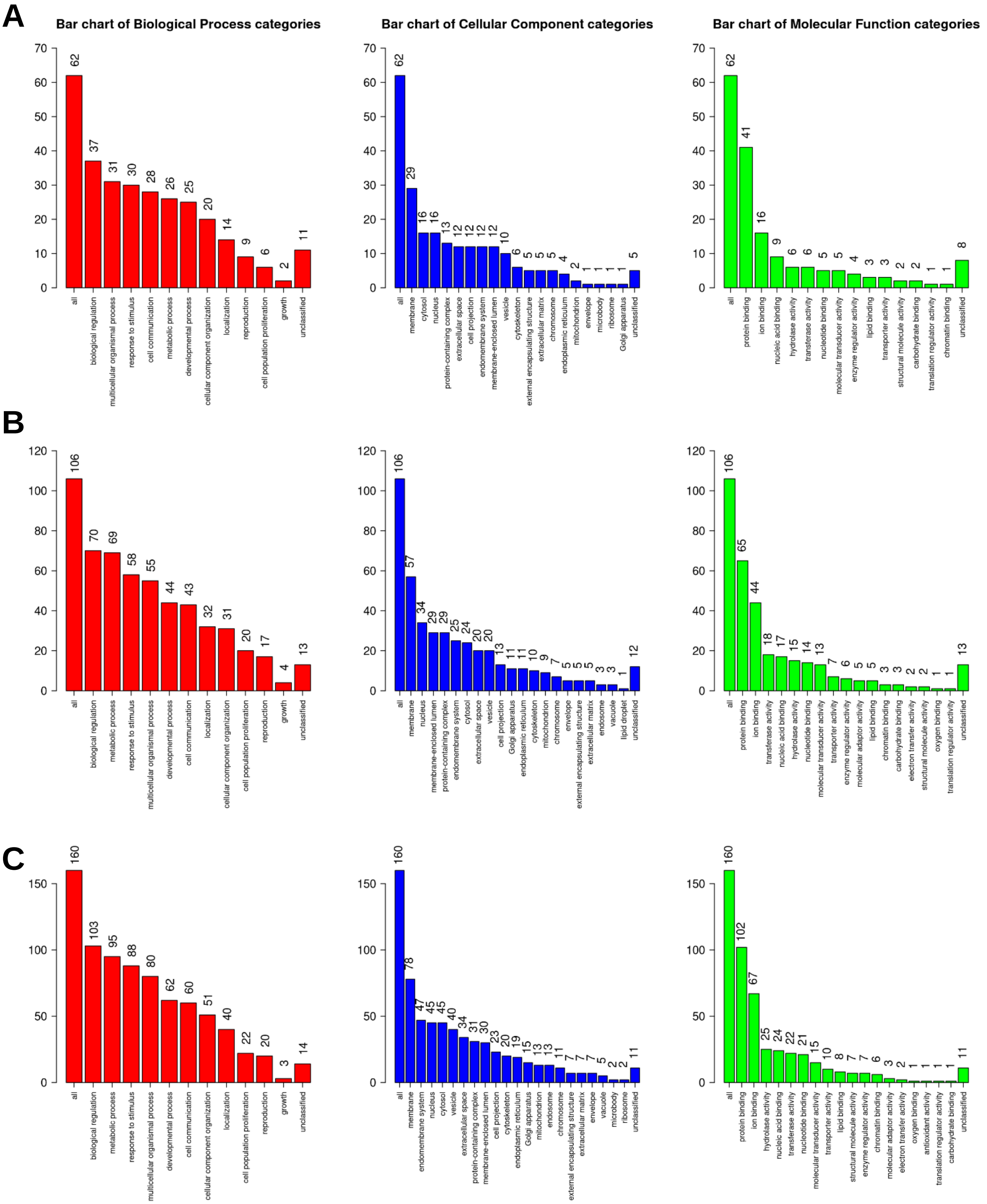

S2 Fig. GO terms for Biological Processes, Cellular Components and Molecular Functions are represented by red, blue and green bars, respectively. A) CBZ, B) VK3 and C) CBZ+VK3. The height of the bar represents the number of IDs in the user list and also in the category.
