# Supplementary material for: In Vitro Effects of Cabazitaxel and Menadione on Cell Growth, Metabolism, and Transcriptomic Profile of Human Prostate Cancer Cell Lines
Source: Prostate Cancer. 2026 May 17;2026:4174599. doi: 10.1155/proc/4174599 (PMC13181216; doi:10.1155/proc/4174599)
Supplement: Supplementary file 3 — Supporting Information 3 Overlapped DEGs in the different contrasts. [file PROC-2026-4174599-s005.pdf]

Overlapped DEGs			
**Upregulated**	**Downregulated**		
**VK3 / VK3+CBZ**	**CBZ / VK3**	**CBZ / VK3+CBZ**	**VK3 / VK3+CBZ**
CYP1A1	OR51G2	FAM110C	CASP14
CYP1B1	C17orf98	CNBD2	CSGALNACT1
TTLL1	EXTL2	ANXA13	ZNF93
AKR1C1	TTLL8	SCL22A9	NRXN3
ANKRD52	WDR66	SIGLEC14	TSSK3
AKR1C3		FAM151B	HSPB7
CYP4F11		ADAMTS12	WFDC8
IDO1		ICAM2	TMLHE
MYO1G		OR13C4	TCP11
IL24		MOS	HSPB8
C4orf26		MTMR7	STK32B

S1 Table.
