# Supplementary material for: In Vitro Effects of Cabazitaxel and Menadione on Cell Growth, Metabolism, and Transcriptomic Profile of Human Prostate Cancer Cell Lines
Source: Prostate Cancer. 2026 May 17;2026:4174599. doi: 10.1155/proc/4174599 (PMC13181216; doi:10.1155/proc/4174599)
Supplement: Supplementary file 4 — Supporting Information 4 Supporting Table S2. Selected DEGs from microarray analysis of CBZ, VK3, and CBZ + VK3 versus CTR. DEG: differentially expressed genes; FC: fold change. [file PROC-2026-4174599-s004.pdf]

Contrast	Gene	FC	p-value
CBZ (UP)	*MSX1*	1.66	1.61E-02
CBZ (DOWN)	*ZRSR2*	0.59	4.67E-02
VK3 (UP)	*CYP1A1*	3.41	6.58E-05
VK3 (DOWN)	*GALNTL6*	0.59	9.63E-04
CBZ+VK3 (UP)	*IL24*	1.67	2.36E-02
CBZ+VK3 (UP)	*IL18R1*	1.72	2.75E-02

S2 Table.
